# ICTV Virus Taxonomy Profile: Matonaviridae 2022

**DOI:** 10.1099/jgv.0.001817

**Published:** 2022-12-20

**Authors:** Annette Mankertz, Min-Hsin Chen, Tony L. Goldberg, Judith M. Hübschen, Florian Pfaff, Rainer G. Ulrich, ICTV Report Consortium

**Affiliations:** 1 Department of Infectious Diseases, Robert Koch-Institute, 13353 Berlin, Germany; 2 Viral Vaccine Preventable Diseases Branch, Centers for Disease Control and Prevention, Atlanta, GA 30333, USA; 3 Department of Pathobiological Sciences, University of Wisconsin-Madison, Madison WI 53706, USA; 4 Department of Infection and Immunity, Luxembourg Institute of Health, L-4354 Esch-sur-Alzette, Luxembourg; 5 Institute of Diagnostic Virology, Friedrich-Loeffler-Institute, 17493 Greifswald-Insel Riems, Germany; 6 Institute of Novel and Emerging Infectious Diseases, Friedrich-Loeffler-Institute, 17493 Greifswald-Insel Riems, Germany

**Keywords:** ICTV Report, *Matonaviridae*, rubella virus, taxonomy

## Abstract

The family *Matonaviridae* comprises enveloped viruses with positive-sense RNA genomes of 9.6–10 kb. The genus *Rubivirus* includes rubella virus (species *Rubivirus rubellae*) infecting humans, ruhugu virus (species *Rubivirus ruteetense*) infecting bats and rustrela virus (species *Rubivirus strelense*) infecting rodents and zoo animals. Rubella virus is spread via droplets. Postnatal infection leads to benign disease with rash and fever. Infection of seronegative women with rubella virus during the first trimester of pregnancy will often result in severe foetal malformations, known as congenital rubella syndrome. Vaccines are globally available. This is a summary of the International Committee on Taxonomy of Viruses (ICTV) Report on the family *Matonaviridae,* which is available at ictv.global/report/matonaviridae.

## Virion

**Fig. 1.**
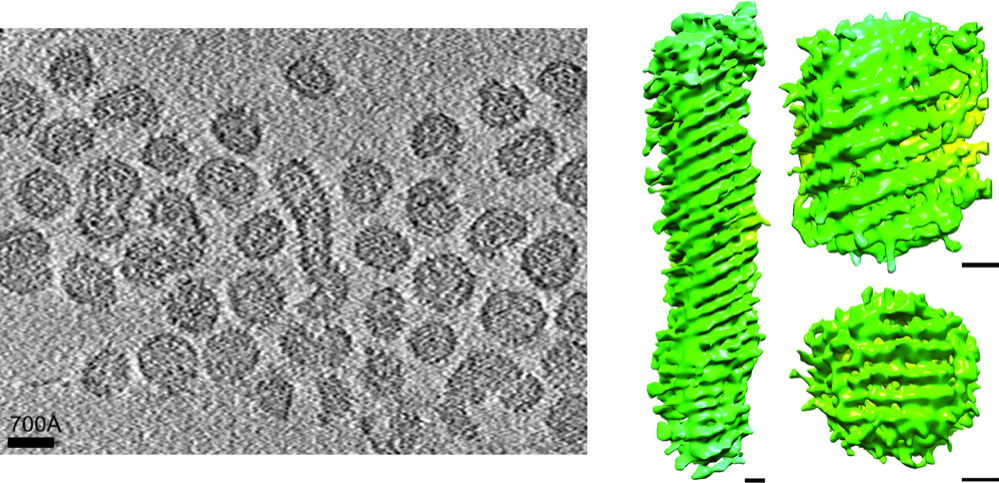
Images and structures of rubella virus particles. (left) Purified rubella virus flash frozen in vitreous ice. (right) Representation of three different rubella virions, each determined using cryo-electron tomography without averaging procedures. Bars: 100 Å. The resolution of the reconstructions are not absolute but are estimated to be better than 50 Å (reproduced from [1]).

Particles of rubella virus are heterogenous, ranging from spherical to tube-like in shape (Table 1, Fig. 1). Particles range between 50 and 90 nm in length and width, with a nucleocapsid core, a lipid bilayer and surface glycoproteins [1].

**Table 1.** Characteristics of members of the family *Matonaviridae*

Example:	rubella virus (JN635296), species *Rubivirus rubellae*, genus *Rubivirus*
Virion	Enveloped, 50–90 nm pleomorphic virions, spherical to tube-like, with a single capsid protein and two envelope glycoproteins
Genome	9.6–10 kb of positive-sense, non-segmented RNA
Replication	Cytoplasmic
Translation	Non-structural proteins are translated from genomic RNA, and structural proteins from subgenomic RNA
Host range	Humans (rubella virus), bats (ruhugu virus), rodents and zoo animals (rustrela virus)
Taxonomy	Realm *Riboviria*, kingdom *Orthornavirae*, phylum *Kitrinovircota*, class *Alsuviricetes*, order *Hepelivirales*: the genus *Rubivirus* includes several species

## Genome

**Fig. 2.**
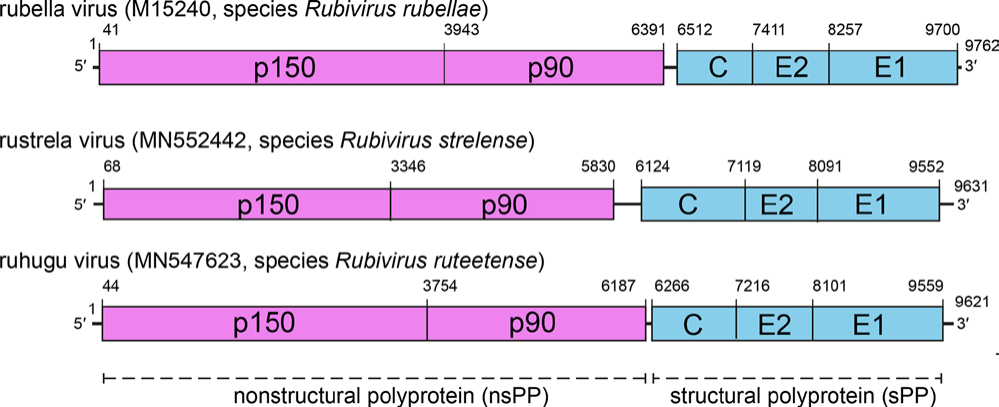
Comparative genome architecture of rubella virus, rustrela virus and ruhugu virus showing the ORFs encoding the non-structural polyprotein (nsPP, pink), the structural polyprotein (sPP, blue), and the boundaries of processed products.

Members of the family *Matonaviridae* have positive-sense RNA genomes of 9.6–10 kb with high G+C contents – 69 % in rubella virus. Genomes possess two open reading frames encoding non-structural proteins and structural proteins (Fig. 2). In addition to the genome RNA, a subgenomic RNA encoding the structural proteins is synthesized during replication. Both the genomic and subgenomic RNAs have a viral type 0 ^7me^GpppA cap at their 5ʹ-terminus and a 3ʹ-non-coding region with poly-A tail at their 3ʹ-terminus [2,3].

## Replication

Rubella virus induces a persistent infection in human foetal endothelial cells [4]. Replication is comparatively slow and, even at a high multiplicity of infection, cells are not uniformly infected after 24 h. Viral replication occurs in association with cellular membranes leading to formation of viral replication factory complexes. For rubella virus, genome replication initiates from the synthesis of negative-sense RNA that is complementary to the genomic sequence. This RNA serves as a template for both genomic and subgenomic RNA synthesis, the latter driven by the subgenomic RNA promoter present in the intergenic region [5]. Two non-structural proteins (p90 and p150) are expressed from the viral genome, while the three structural proteins C, E1 and E2 are expressed from the subgenomic RNA.

## Pathogenicity

The illness produced in humans by rubella virus infection, known as rubella, is generally associated with fever and rash; complications such as arthralgia and arthritis are rare. However, infection of a seronegative woman with rubella virus during the first trimester of pregnancy leads to a >80 % risk of the foetus developing birth defects known as congenital rubella syndrome, including deafness, cataracts and heart defects. Rubella virus is endemic worldwide and is vaccine-preventable [6].

## Taxonomy

Current taxonomy: ictv.global/taxonomy. The genus *Rubivirus* includes the species *Rubivirus rubellae, Rubivirus ruteetense* and *Rubivirus strelense* [7,8]. Rubella virus was previously classified in the family *Togaviridae*.

## Resources

Full ICTV Report on the family *Matonaviridae*: ictv.global/report/matonaviridae
